# CROSS-CULTURAL ADAPTATION TO BRAZILIAN PORTUGUESE OF AN INSTRUMENT TO ASSESS TEASING DURING PHYSICAL/SPORTS ACTIVITY AMONG BRAZILIAN ADOLESCENTS

**DOI:** 10.1590/1984-0462/;2018;36;3;00005

**Published:** 2018

**Authors:** Duana Torquato Dias, Gaia Salvador Claumann, Marina Ribovski, Alexandra Folle, Gelcemar Oliveira Farias, Diego Augusto Santos Silva, Andreia Pelegrini

**Affiliations:** a Universidade do Estado de Santa Catarina, Florianópolis, SC, Brasil.; b Universidade Federal de Santa Catarina, Florianópolis, SC, Brasil.

**Keywords:** Translation process, Adolescent behavior, Bullying, Physical activity, Tradução, Comportamento do adolescente, Bullying, Atividade motora

## Abstract

**Objective:**

To translate and adapt a questionnaire aimed to evaluate teasing during the practice of physical/sports activities in the adolescent population.

**Methods:**

The whole process had six stages: four translations, elaboration of a synthesis version, two back-translations, evaluation by a committee of experts, test of pre-final version with the target population, and presentation of final version to a committee of experts. Thirty-eight adolescents aged between 11 and 18 years participated in the pre-final version test, all of them from the 6th grade of elementary school to senior year of high school in a public school of the state of Santa Catarina.

**Results:**

The steps were strictly followed and then the need for change in the instrument emerged. The questions were altered according to discrepancies observed in back-translations, as well as suggestions by specialists to improve understanding and/or clarity and notes of adolescents participating in the pre-final test.

**Conclusions:**

The instrument was translated into Portuguese and adapted to the Brazilian context according to the reality and culture of adolescents aged 11 to 18 years old, with possible understanding failures in younger age groups.

## RESUMO

**Objetivo:**

Traduzir e adaptar para o português brasileiro um questionário para avaliação das provocações durante a prática de atividades físicas/esportivas para utilização com a população adolescente.

**Métodos:**

O processo foi conduzido em seis etapas: quatro traduções, elaboração de uma versão sintética, duas retrotraduções, avaliação por um comitê de especialistas, teste da versão pré-final com a população-alvo e apresentação da versão final ao comitê de especialistas. Participaram do teste da versão pré-final 38 adolescentes com idades de 11 a 18 anos, estudantes do sexto ano do ensino fundamental ao terceiro ano do ensino médio em uma escola pública de Santa Catarina.

**Resultados:**

As etapas foram seguidas rigorosamente, e a partir delas surgiram necessidades de alteração no instrumento. As questões foram modificadas de acordo com as divergências observadas nas retrotraduções, as sugestões realizadas pelos especialistas para melhoria da compreensão e/ou clareza e baseadas nos apontamentos dos adolescentes participantes do teste da versão pré-final.

**Conclusões:**

O instrumento foi traduzido para o português e adaptado para o contexto brasileiro, conforme a realidade e a cultura de adolescentes de 11 a 18 anos, apresentando dificuldades de compreensão para faixas etárias menores.

## INTRODUCTION

The issue of bullying has been widely discussed in different ways and contexts, such as media and schools.^1^ ^,^ ^2^ This is due mainly to the countless damages inflicted on the victims’ mental and physical health,^3^ ^,^ ^4^ leading them to devaluing feelings, social isolation, and repressed fear or anger.^3^ In addition, bullying has consequences such as social harm, difficulty in concentration,^3^ relationship problems, and hyperactivity.^4^

The internationalization of the concept of bullying, in parallel to the increase in violence, has made it trivial.^5^ However, what happens, mostly among young people, are teasing, conceived as a personal communication that combines elements of humor, aggression, and ambiguity.^6^ Such teasing can take place by comments, laughter or gesture insinuations, often coming from close people such as family and friends.^7^ The aspect of “fun” confused with a hostile act is what distinguishes teasing from bullying.^8^ However, typical teasing behaviors, in addition to being susceptible to aggravation and to becoming bullying, can bring consequences just as severe.

Adolescents are more vulnerable to teasing, especially regarding physical appearance, as adolescence is a period of many changes^9^ associated with, among other facets, the process of biologic maturation.^10^ These changes can cause insecurity especially when individuals do not consider themselves included in ideal physical standards, and thus become vulnerable to negative influences and judgments.^11^

Given the importance of recognizing the scenarios most inclined to teasing, school and sports or physical activities are listed as situations that are most prone to this practice.^12^ ^,^ ^13^ Specifically during physical activity, when one’s bodily performance does not meet required standards, adolescents may be exposed to teasing related to appearance or the way they perform body movements.^14^ This remark supports the statement that body changes during adolescence may interfere with interest for sports and performance,^15^ hence the importance of investigating these behaviors in such contexts. 

In 2011, an instrument was developed in English to evaluate forms of teasing during physical activity among adolescents,^14^ being short and simple tool which covers possible situations related to different aspects, such as physical appearance and motor coordination. Other instruments conceived in English language and aimed at evaluating teasing have been found in literature,^16^ ^,^ ^17^ ^,^ ^18^ ^,^ ^19^ but not intended to the adolescent population only and/or prioritizing physical activity/sports situations. Only one instrument covering this purpose was found, but it was limited to body weight-related teasing among children with mean age of 11.6 (1.24) years and also originally conceived in English language.^20^ Among the instruments mentioned above, two^18^ ^,^ ^20^ were translated, adapted and validated for Brazilian adolescents for a study,^21^ but data regarding this process have not been published.

Aside from few instruments aimed at teasing being found, two studies conducted with adolescents^12^ ^,^ ^22^ (the former with mean age of 14.4 and the latter, 16.4 years) had questions elaborated by the authors of the research instead of validated questionnaires. It is worth mentioning that there are no Brazilian instruments made public or any material adapted to the Brazilian Portuguese language, which possibly led researchers to use instruments literally translated, and therefore to misconceptions, since the cultural reality of the original tool was not adapted to the population in question.

In view of the above, this study proposes an important initiative, because an instrument to evaluate teasing, negative comments and experiences of adolescents during the practice of physical/sports activities, translated and adapted for the Brazilian population is of great value for both professionals who deal with this group of people and may face similar situations of teasing, and therefore can manage to prevent adolescents from causing psychological damages to others or situations of aggravation (bullying); it might also guide health researchers, as they will have an additional tool for studies on this subject at their disposal. 

In this perspective, this study’s purpose was to translate and adapt to Brazilian Portuguese a questionnaire assessing teasing during the practice of physical/sports activities to be used with the adolescent population.

## METHOD

The original instrument is an English-language questionnaire^14^, composed of four questions developed to evaluate negative experiences, comments, and teasing among adolescents aged 12 to 16 years who practice sports or physical activities. In addition, the questionnaire has a second section in which the adolescents can choose from five alternatives the individual(s) who is held responsible for such negative experiences.

The method by Beaton et al.^23^ was used in the cross-cultural adaptation of the original instrument into Brazilian Portuguese, consisting of six steps:

1. initial translation of the original instrument by at least two independent translators;
2. synthesis of translations;
3. back-translation;
4. evaluation by a committee of specialists;
5. testing of pre-final version with the target population and;
6. referral of documentation to the committee of specialists.

The original instrument was independently translated into Portuguese by four Brazilian professionals, all fluent in English. In the second step, the four translations were compared by three researchers of the study and synthesized in a single version.

The third stage was carried out by two professionals from English-speaking countries and fluent in Portuguese, who back-translated the synthesis of translations into the official language. Both independent back-translations were compared with one another and with the original instrument to check that the translated version was transmitting the primary concept of the instrument.

Subsequently, a document containing the original questions of the instrument and the Portuguese version was referred to a committee of specialists, formed by five higher-education professors in the areas of Physical Education and Pedagogy, all fluent in English, so they could evaluate it as to semantic (transfer of meaning between languages); idiomatic (equivalent colloquialisms in both languages); experimental (situations covered should match with Brazil’s cultural reality); and conceptual equivalence (the document can be well understood and used in a universal way). 

Reports on the synthesis of translations and changes made after back-translations were also included in the document. The evaluation was based on an ordinal scoring: 1- inadequate, 2- somewhat adequate, 3- acceptable, 4- adequate,5- very adequate. For scores 1, 2 or 3, the evaluator was requested to leave an amend suggestion.

The version of the questionnaire modified after the specialist’ evaluations (pre-final version) was tested with the target population, consisting of 38 adolescents (20 girls and 18 boys) aged 11 to 18, attending from 6th grade to the 3rd year of high school at a state public school of Santa Catarina. A drawn was made to select a class per grade. The research leaders explained the purpose of the study to groups selected, inviting them to voluntarily answer the questionnaire and participate in the interview. Those who agreed to participate signed an informed consent form and, when underage, they were supposed to hand another consent form to their parents/caregivers, so they would take notice and authorize their participation. The adolescents aged 18 or more signed their own consent form

In order to collect data, adolescents were informed about the purpose of the instrument and that they could withdraw from the research at any moment without loss. Later, separated by class, they answered the questionnaire individually within ten minutes on average. Afterwards, researchers conducted an interview with all adolescents, questioning their understanding of each question, possible doubts, and difficulties faced, as well as criticisms and suggestions regarding the content and the filling in of the forms, each talk having lasted approximately 15 minutes. All interviewees attended the classes of Physical Education and some of them also practiced extracurricular physical or sports activities. 

After pre-test, the final version of the questionnaire was sent to the committee of specialists so they were aware of the results. In addition, the researchers contacted the participants again with the pre-final version of the modified questionnaire after this stage, verifying suitability and thus establishing the final version.

This cross-sectional study is part of a macro project entitled “Brazilian Guide for the Evaluation of Health-related Physical Fitness and Life Habits - Stage I”, approved by the Research on Human Being Ethics Committee s of Universidade Federal de Santa Catarina (Protocol 746,536 / 2014).

To complete the procedures of translation and adaptation of this instrument related to teasing during the practice of sports or physical activities, consent and authorization were obtained from one of the proponents via e-mail.

## RESULTS

**Chart 1:**
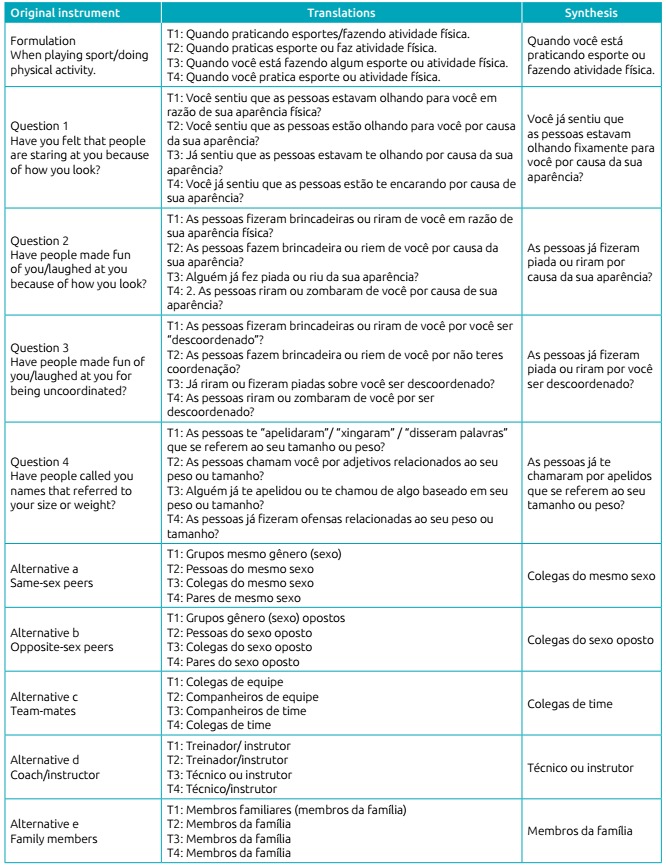
Original instrument, translations into Brazilian Portuguese (T1, T2, T3 and T4), and synthesis of translations.

The instrument in its original version, the four translations into Brazilian Portuguese (T1, T2, T3, and T4) and the synthesis of translations are presented in Chart 1.

In the first stage of the cross-cultural adaptation process, four versions of the original instrument were obtained after translation into Brazilian Portuguese, being afterwards compared and synthesized in one version. In order to establish this version, words translated equally in most versions were maintained, while divergent translations were debated between researchers to define the best suitable option, according to the instrument’s objectives.

In the first sentence of the instrument, the expression “quando você está praticando” (which means, “when you are practicing”) was chosen, which means that questions addressed the moment of physical/sports activity practice.

In question number 1, the researchers preferred “você já sentiu” (“have you ever felt”) to “você sentiu” (“have you felt”), even when the latter appeared in two of the four translations, since it could give the adolescent the option of one occasion only, when the intention is to think of all times it has happened. The same criterion led the researchers to decide upon the expression “estavam olhando” (which means, “they were looking”) in the same question.

Questions 2 and 3 start the same (“Have people made fun of you/laughed at you”) and both have quite different translations. The expression “já fizeram” (“has anyone made”) was used to refer to any occasion experienced and the word “piada” (“joke”) was considered more appropriate than words related to “brincadeira” (“play”) and “zombaram” (“mocking”).

In question 4, also to refer to all situations of teasing ever experienced, the word “já” (“already”) was retained in the sentence “as pessoas já te chamaram” (which means, “have people ever called you”) and, further, researchers considered the term “apelidos” (“names”) more appropriate than “adjetivos” (“adjectves”), as well as more subtle than “fizeram ofensas” (“offended”) and “xingaram” (“cursed”).

In the four alternatives related to the individual(s) responsible for teasing, alternatives “a” and “b” had four different translations for its first word. For better understanding, the word “colegas” (which means, “classmates”) was defined in both. In alternative “c”, “colegas de time” (“teammates”) was preferred because it seemed more suitable to sports practice. Alternative “d” was equally translated in all versions, thus remaining the same. Alternative “e” had three identical translations which were then maintained.

**Chart 2:**
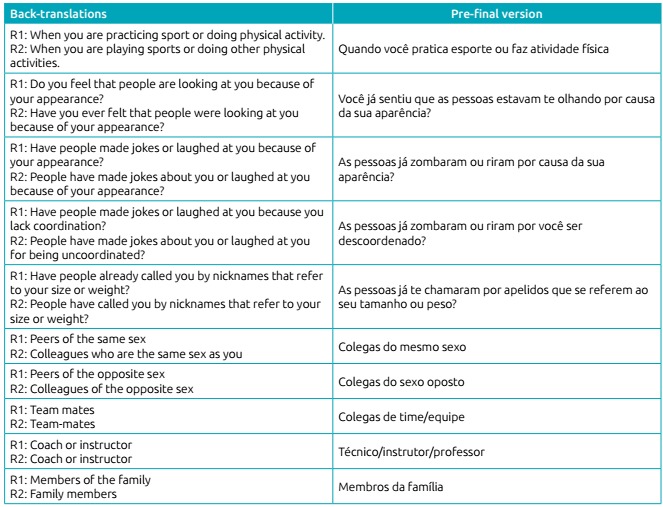
Back-translations (R1 and R2) and pre-final version of the instrument.

The back-translations (R1 and R2) and the pre-final version in Portuguese are presented in Chart 2.

In the phase of back-translation, the documents were compared to the original instrument to check for possible meaning discrepancies in the adaptation, when three sentences were modified.

In the first question, the word “fixamente” (“fixedly”) was added to emphasize that it was not referring to a quick glance, but to a more intense way of looking. In the second and third questions, the words “fizeram piadas” (“made fun”) were replaced by “zombaram” (“mocked”), because in back-translation it was translated as “made jokes”, which distances the meaning a lot from the aim of the original instrument.

After adjustments, the instrument was forwarded to a committee of specialists with fluency in English language: a Ph.D in Education and four Ph.D in Physical Education. Three of them were experienced in School Physical Education, School Sports and Brazilian Education.

The original instrument had no instructions on how questions should be answered. Therefore, statements were elaborated by the researchers and referred to in this document for the specialists to check them for clarity. Two reports were attached to the document: the synthesis of translations and the changes made after back-translations.

The suggestions made by specialists resulted in six modifications. The first sentence of the instrument was questioned as to its verb tense. According to them, the English original version was in the simple present and the translated version was in the present continuous, that is, it was changed from “quando você pratica esporte ou faz atividade física” (which means, “when you play sports or do physical activity”) to “quando você está praticando esporte ou fazendo atividade física” (which means, “when you are playing sports or doing physical activity”). In question 1, the removal of the word “fixamente” (“fixedly”) was suggested because the experts pondered that the adolescents would not have understand it appropriately. In addition, the expression “olhando para você” (“looking at you”) was changed to a more usual version, “te olhando” (“staring at you”). Therefore, the question was “Você já sentiu que as pessoas estavam te olhando por causa da sua aparência?” (which means, “Have you ever felt that peoples were staring at you because of your appearance?”)

In alternative “c” of question addressing people responsible for the teasing acts, the concept of “equipe” (“team”) was added for a better understanding by the adolescents, changing to “colegas de time/equipe” (“teammates/team”). In this section, also about possible bullies, in alternative “d”, the specialists noticed that in the Brazilian physical education classes or in sports schools the term “instrutor” (“instructor”) was rarely used, so they suggested replacing it or adding the term “professor” (“teacher”). In order to encompass all physical and/or sports activity situations, it was decided to add the term. Therefore, the alternative became “técnico/instrutor/professor” (“coach/instructor/teacher”).

Regarding the statements of the questions, changes were suggested for both. In the first, it was recommended to add a caption for each number of the response scale, since in the forwarded version there were captions only in the first and last number and teenagers might not understand the intermediate options. Thus, the caption of “1= nunca, 2, 3, 4, 5= frequentemente” (which means, 1= never, 2, 3, 4, 5=frequently) was altered to “1= nunca, 2= quase nunca, 3= às vezes, 4= quase sempre, 5= sempre” (“1= never, 2= hardly, 3= sometimes, 4= often, 5= always”). 

The second statement had a textual simplification as a suggestion, modifying from “Caso você tenha respondido alguma opção diferente de “1 (nunca)” nas questões acima, assinale abaixo quem foi o responsável por este comentário/experiência (obs.: se existirem, você pode assinalar mais de uma opção)” (which means, “In case you have chosen any option other than “1 (never)” in the above questions, check below who the responsible for this comment/experience was (note: you may check more than one option)”) to “Caso você tenha respondido, em alguma das questões acima, as opções de 2 (quase nunca) a 5 (sempre), indique quem foi o responsável por este comentário/experiência. Obs.: se o comentário ou experiência teve a participação de mais de uma pessoa, você pode assinalar mais de uma opção”. (which means, “If you have chosen option 2 (hardly) to 5 (always) in any of the above questions, indicate who was responsible for this comment/experience. Note: if the comment or experience involved more than one person, you can check more than one option.”). The modifications suggested by the specialists were applied to the pre-final version for testing.

The adolescents understood the first sentence of the questionnaire as an additional question, so they proposed a change of placement, and most suggested placing it next to the statement. Therefore, the statement was amended to “Responda as questões abaixo, pensando em quando você pratica esporte ou faz atividade física, assinalando uma das opções da escala, e considerando a seguinte legenda” (“answer the questions below, thinking about when you play sports or do physical activity, checking one of the options according to the following caption.”)

In questions 1 and 2 the word “aparência” (“appearance”) was widely interpreted. The adolescents considered “aparência” (“appearance”) as a way of dressing, arranging the hair, the way of walking, among others. The purpose of the original questionnaire was to refer only to physical appearance (body shape, type, and dimensions). Participants suggested that the appropriate term would be “aparência física” (“physical appearance”), rather than “aparência” (“appearance”) only. Thereby, the term was replaced in both questions.

In question 3, most adolescents had difficulty understanding the term “descoordenado” (“uncoordinated”). They recommended an insertion of examples or explanations alongside the term, to help in understanding. That way, the question was changed to “As pessoas já zombaram ou riram por você ser descoordenado (por exemplo: ter dificuldade para realizar as atividades físicas, ou realizá-las de maneira desajeitada)?” (which means, “Have people mocked or laughed at you for being uncoordinated (for example: having difficulty performing physical activities, or performing them clumsily)?”).

The word “turma” (“class”) was added to alternative “c”, which encompasses the possibilities of people responsible for the teasing, as most individuals referred only to their team players and the purpose of the questionnaire is to include the larger group. In alternative “e”, an example of family members was added because some adolescents understood family members only as father, mother, and siblings. This alternative was modified to “membros da família (por exemplo: pai, mãe, irmãos, tios, avós, primos...)” (which means, “family members (e.g.: father, mother, siblings, uncles, grandparents, cousins...)”).

**Chart 3:**
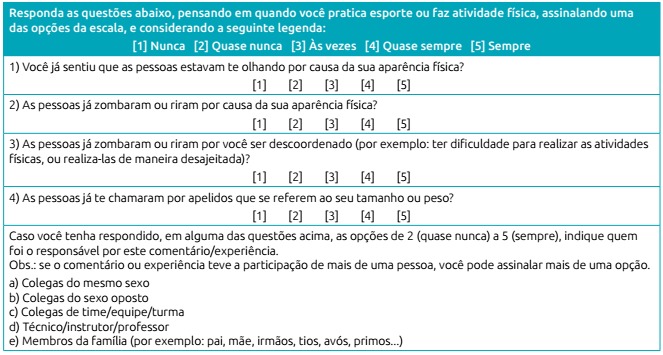
Final version of the questionnaire on teasing during physical activity.

The final version of the questionnaire, after suggestions by adolescents were applied, is presented in Chart 3.

## DISCUSSION

The process of cross-cultural adaptation to Brazilian Portuguese was carried out systematically and strictly,^22^ presenting satisfactory results. The method proposed by Beaton et al.^23^ has very important adaptation stages, aiming at equivalence of meaning, colloquialisms, target language speakers, and their possible background. Following the steps of the method defined is of supreme importance, so that the final version of the instrument is truly adequate for the population in question.^24^

In the stages of translation and back-translation, Beaton et al.^23^ point out the need for more than one translator. This multiplicity enabled wide debates about some disparities between suggestions of modifications, in order to obtain the word or expression more suitable to the Brazilian culture and maintaining the purpose of the original instrument.

That being done, a document was sent to the committee of specialists, emphasizing equivalence adaptations. The expertise of the experts committee allowed important adjustments to terms so they would represent the original’s sense and could be applied to the target population.

The test with the pre-final version was destined to check that the instrument was understandable by its target audience. The emphasis of this phase was brought to attention because, although the questionnaire appeared to be ready, there were terms that were difficult for adolescents to understand, and subtle suggestions of changes made by them made the instrument clearer in the final version.

Younger individuals (11-12 years old) had greater difficulties understanding the questions in the context of physical and/or sports activity practice, having reported situations occurred in contexts other than these, for example at home, in a meeting with friends and/or in the classroom. Thus, even when the instrument is adapted, the validation process is of extremely importance to verify its validity to younger age groups.

Cross-cultural adaptation is the first step in validating an instrument.^23^ Studies investigating psychometric properties (reproducibility, internal consistency, external validity) are still required for the Questionnaire for Assessment of Teasing During the Practice of Physical Activities to be used in research with the adolescent population in Brazil.

The questionnaire was translated into Portuguese and adapted to the Brazilian context, according to the reality and culture of adolescents aging between 11 and 18 years old, with understanding problems when it came to younger age groups.
